# Design and Preliminary Evaluation of a Community-Based Brain Health Promotion and Wellness Program

**DOI:** 10.1093/geroni/igaa057.724

**Published:** 2020-12-16

**Authors:** Laura Block, Megan Peck, Andrea Gilmore-Bykovskyi

**Affiliations:** 1 University of Wisconsin-Madison School of Nursing, Madison, Wisconsin, United States; 2 University of Wisconsin-Madison, Madison, Wisconsin, United States

## Abstract

Recommendations for risk reduction of dementia and cognitive decline emphasize addressing modifiable risk factors including physical activity, cognitive stimulation, and socialization. However, existing resources and programs to promote brain health through multifaceted risk factor reduction are often costly, challenging to individualize, and limited in their delivery format, narrowing their accessibility among older adults. To address community identified needs for brain health promotion resources, we applied a user-centered design approach to develop, implement, and evaluate feasibility/acceptability of a community-based brain health promotion program: Strength & Resilience Brain Health and Wellness Program. Design requirements were specified through ongoing end-user feedback via structured and open-ended surveys, group dialogue, and facilitator memoing which informed needed iterative refinements of program components. The resultant program incorporates information about brain health/dementia prevention, physical exercise, and cognitively-stimulating activities targeting attention, focus, problem solving, and communication. Integrated across components are deliberate adaptations for physical/cognitive abilities and activities to facilitate group cohesion and reduce stigma. Across two 10-week offerings, we found the program to be feasible as demonstrated through high enrollment (N=44) and retention (75%). A quarter of participants self-disclosed a dementia diagnosis in baseline surveys. Participants rated program components as acceptable, and perceived positive influences on target risk factors particularly social isolation, sense of self-worth, and cognitive stimulation. The current study provides a platform for a feasible and scalable group, community-based brain health promotion program, and suggests participant-centered outcomes extend beyond cognitive domains. More rigorous evaluation of the program is needed to evaluate fidelity across components and standardized outcomes.

